# Elucidation of Complex Nature of PEG Induced Drought-Stress Response in Rice Root Using Comparative Proteomics Approach

**DOI:** 10.3389/fpls.2016.01466

**Published:** 2016-09-29

**Authors:** Lalit Agrawal, Swati Gupta, Shashank K. Mishra, Garima Pandey, Susheel Kumar, Puneet S. Chauhan, Debasis Chakrabarty, Chandra S. Nautiyal

**Affiliations:** Division of Plant Microbe Interactions, Council of Scientific and Industrial Research-National Botanical Research InstituteLucknow, India

**Keywords:** drought, proteomics, rice root, tolerant cultivar, 2-DE, MALDI-MS/MS, SOTA analysis

## Abstract

Along with many adaptive strategies, dynamic changes in protein abundance seem to be the common strategy to cope up with abiotic stresses which can be best explored through proteomics. Understanding of drought response is the key to decipher regulatory mechanism of better adaptation. Rice (*Oryza sativa* L.) proteome represents a phenomenal source of proteins that govern traits of agronomic importance, such as drought tolerance. In this study, a comparison of root cytoplasmic proteome was done for a drought tolerant rice (Heena) cultivar in PEG induced drought conditions. A total of 510 protein spots were observed by PDQuest analysis and 125 differentially regulated spots were subjected for MALDI-TOF MS-MS analysis out of which 102 protein spots identified which further led to identification of 78 proteins with a significant score. These 78 differentially expressed proteins appeared to be involved in different biological pathways. The largest percentage of identified proteins was involved in bioenergy and metabolism (29%) and mainly consists of malate dehydrogenase, succinyl-CoA, putative acetyl-CoA synthetase, and pyruvate dehydrogenase etc. This was followed by proteins related to cell defense and rescue (22%) such as monodehydroascorbate reductase and stress-induced protein sti1, then by protein biogenesis and storage class (21%) e.g. putative thiamine biosynthesis protein, putative beta-alanine synthase, and cysteine synthase. Further, cell signaling (9%) proteins like actin and prolyl endopeptidase, and proteins with miscellaneous function (19%) like Sgt1 and some hypothetical proteins were also represented a large contribution toward drought regulatory mechanism in rice. We propose that protein biogenesis, cell defense, and superior homeostasis may render better drought-adaptation. These findings might expedite the functional determination of the drought-responsive proteins and their prioritization as potential molecular targets for perfect adaptation.

## Introduction

Rice (*Oryza sativa* L.) is an important staple cereal crops ranked second after maize worldwide and considered as a primary source of food for more than half the world's population including Asia. India is the second largest producer of rice after China and account for about 20% of all world rice production. Rice is exclusively grown for human consumption and therefore there is immense pressure for high production to feed the largely growing world population (Ahsan et al., 2008). Climate related diverse abiotic stresses (drought, flood, salt, cold etc.) are the principal sources of risk and uncertainties in agriculture and causes wide fluctuations in agricultural output. While attempts have been made to reduce the adverse effects of weather on agriculture through scientific research and technology development, the performance of agriculture, especially in developing countries, still depends largely on the weather. Drought is the major abiotic stress limiting the productivity of rice as it hampers plant growth and development, and shrinks harvest size (Subba et al., 2013). In Asia, drought, a major abiotic stress, is responsible for affecting about 20% of the total rice-growing area (Pandey and Bhandari, 2008). Rice germplasm exhibit a tremendous genetic source for controlling agronomical importance characters such as drought tolerance. Moreover, relatively smaller genome size and well-organized database make it a choice of model plant for monocots to study the physiological, biochemical and molecular aspects during development, abiotic as well as biotic stress conditions (Atkinson and Urwin, 2012; Narsai et al., 2013). The better understanding of drought tolerance mechanism and the efficiency to develop drought tolerant varieties can be correlated with identification of trait associated genes (Tuberosa and Salvi, 2006; Lafitte et al., 2007; Sreenivasulu et al., 2007). However, it remains a challenging task due to its highly complex network of several metabolic pathways (Price et al., 2002). Understanding the mechanism of dehydration response is the key to decipher the regulatory mechanism of better adaptation (Subba et al., 2013).

Plant roots are important organs to uptake soil water and nutrients, perceiving and transducing of soil water deficit signals to shoot (Davies and Zhang, 1991; Moumeni et al., 2011) which further triggers an array of physiological, morphological and molecular responses in the whole plant. Understanding the root responses through transcriptomics with parallel insights on protein level changes has always been an area of interest and mostly relies on comparative studies of diverse genetic background under drought (Sengupta et al., 2011). Roots are supposed to be the primary site for sensing drought stress to initiate signaling cascade at molecular level in responses to drought. Plants can successfully adopt a tolerance mechanism against environmental stress by regulating gene expression through transcriptional changes in regulatory and functional proteins (Périn et al., 2007). In a study, Kawasaki et al. (2001) observed that gene expression profiling of rice under salt stress may result in up and downregulation of ~10% of the transcripts. However, the gene expression studies do not offer insights into the quantity and quality of the proteins as the amount of proteins is not always correlated to that of mRNA. The post-translational modifications like phosphorylation, glycosylation and removal of signal peptides enable proteins for activities and subcellular localization (Kawasaki et al., 2001). As a consequence, understanding the stress mechanism at protein level may provide some more useful insights to study the actual biotic stress response. In this context, evolution of proteomics is playing a crucial role as necessary and complementary approach to address such issues in the post-genomic era (Zivy and de Vienne, 2000; van Wijk, 2001). Serious effort has been made for functional identification of tissues, organs, and development specific rice gene under environmental changes like biotic and abiotic stresses using systematic studies in proteomic analysis (Khan and Komatsu, 2004; Komatsu, 2005; Ali and Komatsu, 2006) and knowledge of these proteins would help in understanding the stress tolerance related molecular mechanism in rice at the translation level.

We report here the comparative proteomic analysis of rice root for PEG simulated drought responsiveness in time dependent manner. Proteins were separated by two-dimensional gel electrophoresis (2-DE) followed by PD Quest analysis and identified by MALDI-Mass Spectrometry (MS) using available proteome databases.

## Materials and methods

### Plant material collection

After screening of several local rice varieties, a drought tolerant variety Heena cultivated in northern Indian was selected for the present study. Three-weeks-old seedlings of rice were stressed for 7 days using 20% polyethylene glycol PEG-6000 in the nutrient solution. Exposure to PEG-6000 solutions is supposed to mimic drought stress with limited metabolic interferences. Effective screening of large sets of germplasm for drought tolerance has been carried out by PEG-based *in vitro* screening as a suitable method with good accuracy (Muscolo et al., 2014). Roots samples from three independent biological replicates were harvested on first, third, and seventh day and immediately flash frozen in liquid nitrogen and stored independently at −80°C for further analyses.

### Extraction of rice root proteins and 2-dimensional gel electrophoresis (2DE) analysis

Rice roots harvested in three replications were pooled to normalize the effect of variations in the biological replicates and used for extraction of soluble proteins. Root tissue (5 g) was pulverized to a fine powder in liquid nitrogen and suspended in 10 ml lysis buffer containing 0.5 M Tris HCl (pH 7.5), 0.1 M KCl, 50 mM EDTA (pH 8.0), 0.7 M Sucrose, 10 mM thiourea, 2 mM PMSF, and 2% β-mercaptoethenol. Suspension was mixed with 10 ml of Tris saturated phenol (pH 8.0) for 30 min with gentle shaking and centrifuge at 9000 × g for 10 min. Upper phase was transferred in separated vial and 0.1M ammonium acetate (dissolved in methanol) added in upper phase and incubated at −20°C for overnight. Next day, the solution was centrifuged again at 9000 × g and supernatant was discarded. Pellet was washed with 100% chilled acetone twice and then air dried pellet was dissolved in rehydration buffer [8 M urea, 2 M thiourea, 4% (w/v) CHAPS, 20 mMDTT, 0.5% (v/v) Pharmalyte (4–7)]. Total root protein concentration was determined by Bradford assay (Bio-Rad, USA). Isoelectric focusing was carried out with 250 μg of protein as given by Agrawal et al. (2008). Briefly, the 13-cm IEF strips (pH 4–7) were rehydrated with protein for 16 h and electrofocused using an IPGphor system (Bio-Rad, USA) at 20°C up to 25,000 Vh. This focused strips were subjected to reduction with 1% (w/v) DTT in 10 mL of equilibration buffer [6 M urea, 50 mM Tris-HCl (pH 8.8), 30% (v/v) glycerol and 2% (w/v) SDS] and followed by alkylation using 2.5% (w/v) iodoacetamide in same equilibration buffer. The strips were then loaded on top of 12.5% polyacrylamide gels for SDS-PAGE. The gels were fixed and stained with a silver stain plus kit as per protocol (Bio-Rad, USA).

### Image acquisition and data analysis

The silver stained gel obtained after 2DE were scanned for image acquisition using the Bio-Rad FluorS system equipped with a 12-bit camera. Images from three biological replicate 2-DE gels were taken for the data analysis in PDQuest version 8.0.1 (Bio-Rad). The parameters like protein spot quality, molecular mass, and pI of individual protein was assessed as described earlier by Agrawal et al. (2013). The spots showing reproducibility in quality and quantity in at least two of the three replicate gels were taken in consideration during analysis. A normalized spot volume for protein quantification was obtained by PDQuest software using the total spot volume normalization procedure to avoid experimental variations in gels due to protein load or staining.

### Protein identification

The silver stained protein spots were digested for MALDI MS/MS were digested with trypsin (Promega Corporation, MA, USA) as per manufacturer instruction. The stained protein spots were excised manually, washed thoroughly with water and crushed in small pieces. These gel pieces were washed with 15 mM potassium ferricynide and 50 mM sodium thiosulfate (1:1) for destaining. Then gel pieces were dehydrated with solution A [100% Acetonitrile (ACN): 50 mM Ammonium bicarbonate (ABC) in 2:1], subsequently rehydrated with 25 mM ABC. These steps were repeated until gel pieces become white. Final supernatant was discarded and gel pieces were dried in speedvac. After this gel pieces were rehydrated with 0.2 μg/μL trypsin and 50 mM ammonium bicarbonate and incubated at 37°C overnight for digestion. Peptides were extracted from gel slices with 1% TFA in 50% ACN twice. The peptide solution was concentrated down to 5 μL by vacuum centrifuge. Peptide solution mixed with 5 mg/ml CHCA (α-Cyano-4-hydroxycinnamic acid) matrix and spotted onto MALDI plate.

### MALDI-TOF-TOF analysis

A 4800 Proteomics Analyzer (Applied Biosystems, USA) with TOF/TOF optics was used for all MALDI-MS and MS/MS applications. Samples for MALDI were prepared by adding 0.5 μl matrix solution (5 mg/mL a-Cyano-4-hydroxycinnamic acid in 50% ACN containing 0.1% TFA) to 0.5 μl trypsin digested protein sample and left for air dry at room temperature on stainless steel 384 well-target plate after spotting. The plate containing spot was inserted in the mass spectrometer and subjected to mass analysis. A mixture of four proteins angiotensin I, Glu-fibrino-peptide B, ACTH (1e17), and ACTH (18e39) was used to calibrated the mass spectrometer externally. Further, the instrument was externally calibrated with fragment of Glufibrino-peptide B for MS/MS experiments.

The monoisotopic peptide masses obtained from MALDI-TOF/TOF were analyzed by the 4000 Series Explorer software version 3.5. Spectra were collected in a data dependent mode. For each spot total 500 laser shots were accumulated to generate ions for MS analysis and 30 most abundant ions were used for MS/MS analysis. All exported spectra as Mascot Generic Format (MGF) files from MALDI platforms were acquired in linear positive mode, smoothed by the Savitzky–Golay algorithm and the baseline subtracted for further analysis. Peak detection criterion for peak to be considered was set to minimum S/N = 10. Protein identification was performed using Mascot software (http://www.matrixscience.com) NCBInr databases 20160522 (87959973 sequences; 32280238673 residues). The database search criteria were as follows: taxonomy, *O. sativa* (rice) (172275 sequences), peptide tolerance, ±100 ppm, MS/MS tolerance, ±0.2 Da; peptide charge +1; maximum allowed missed cleavage, 1; fixed modification, cysteine carbamidomethylation; variable modification, methionine oxidation; instrument type, MALDI-TOF/TOF. Protein scores were measured from sum of the series of peptide scores as a non-probabilistic basis for ranking protein hits. The only protein spots whose MOWSE score was above the significant threshold level (Table S3) determined by Mascot along with the number of peptides matched and % coverage of matched protein were considered to represent a positive identification. In all the protein identifications, probability scores were greater than the score fixed by Mascot as significant with a *p* < 0.05.

The similar expression pattern of the identified differential proteins was determined by SOTA (self-organizing tree algorithm) clustering on the log transformed fold induction expression values using Multi Experiment Viewer (MEV) software (The Institute for Genomic Research, TIGR) of rice. The clustering was performed with Pearson correlation as distance with 10 cycles and maximum cell diversity of 0.8 (Romijin et al., 2005). SOTA is a neural network that grows adopting a binary tree topology, where hierarchical cluster are obtained as result with the accuracy and robustness (Herrero et al., 2001). Each branch summarizes the patterns of all similarly expressed proteins with centroid expression profile of a group.

### Expression analysis of some selected candidate genes using qRT PCR

Real time-PCR was performed in 20 μl for a set of selected genes using Fast SYBR Green PCR Master Mix (Agilent Technologies, USA). The list of selected genes and oligonucleotide primers (Sigma-Aldrich, USA) used for each gene are listed in Table S1. Oligonucleotide primers for vetiver ubiquitin gene were used as the internal control for establishing equal amounts of cDNA in all reactions. The reactions were performed using the following cycle conditions, an initial 94°C for 2 min, followed by 30 cycles of 94°C for 30 s, 60°C for 30 s, and 72°C for 30 s, and the final 5 min extension at 72°C. After obtaining the ct-value for each reaction, the relative expression was calculated by 2^-delta Ct method.

## Result and discussion

### Dehydration-induced changes and 2-DE analysis

**Figure 1:**
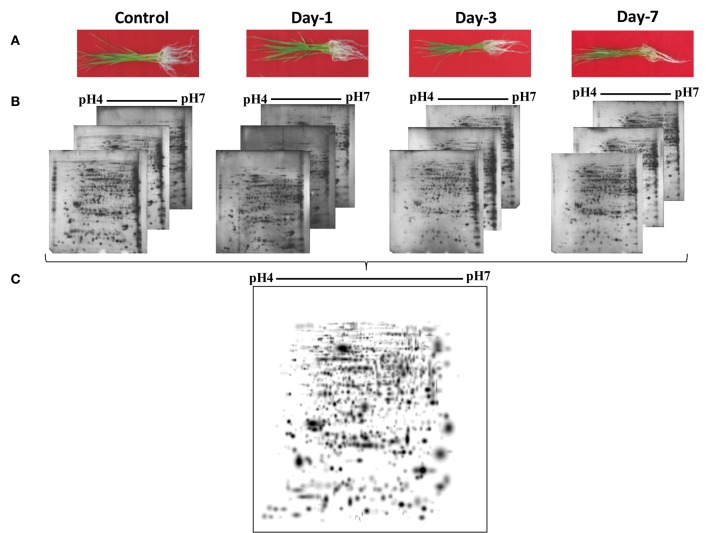
**2-DE analysis of root proteome of rice. (A)** Rice plant showing different stages of drought. **(B)** Proteins were extracted from rice root tissue and equal amounts (250 μg) of proteins were separated by 2-DE as described in Materials and Methods Section. **(C)** Three replicate silver-stained gels for each stage were computationally combined using PDQuest software and one representative master standard gel image was generated.

**Figure 2:**
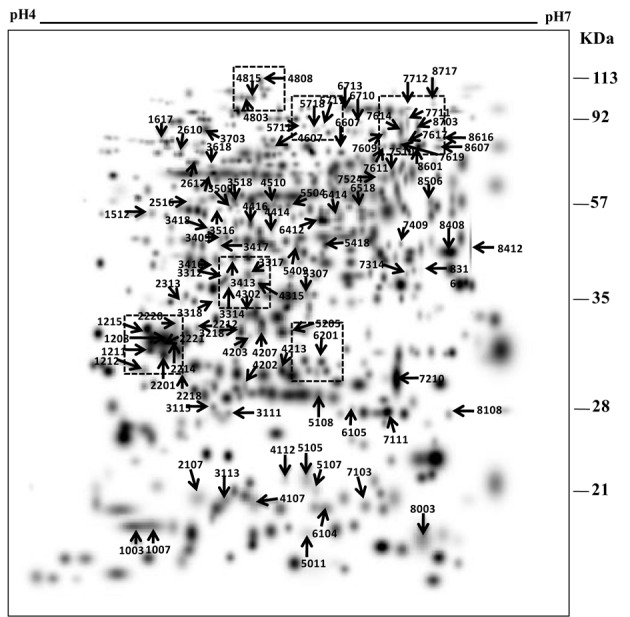
**A higher level master image was created *in silico* by PDQuest from three replicate gels of each time point**. The boxed areas marked with dotted lines represent the zoomed-in gel sections in Figure 3. The numbers correspond with the spot IDs listed in Table 1.

**Figure 3:**
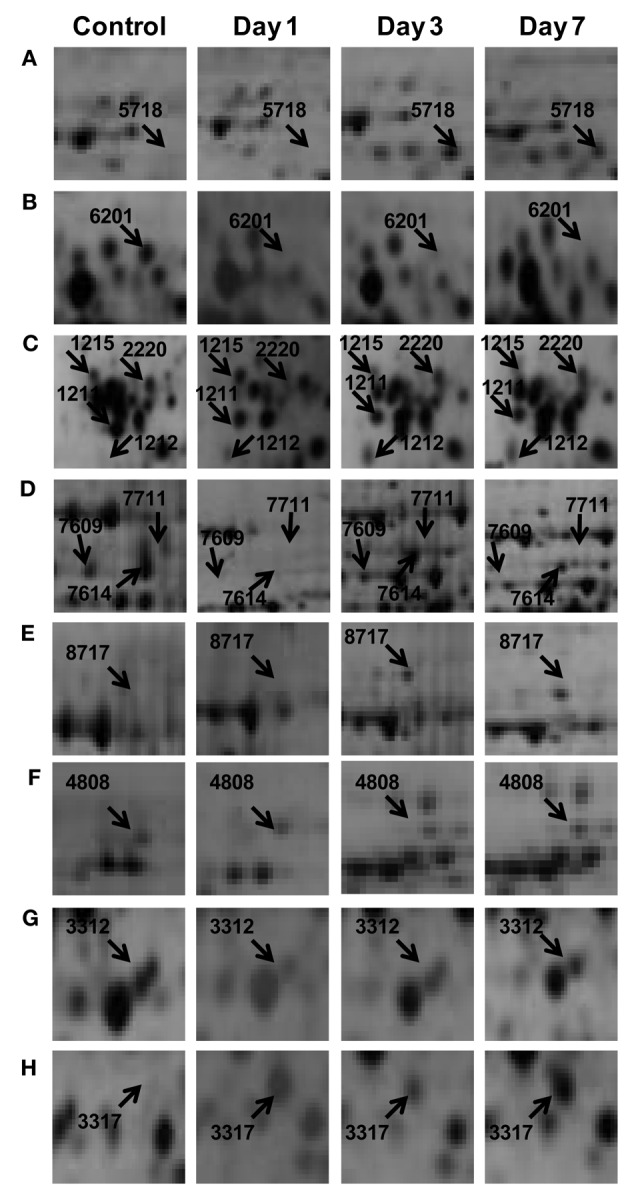
**Some of the differentially expressed root proteins represented in the enlarged gel sections (A–H) corresponds to the marked boxed areas in Figure 2**.

To understand the drought tolerance mechanism in rice, we carried out the comparative proteomic analysis of roots of the drought tolerance Heena cultivar of rice at various time points after drought induction in 10% PEG (Figure 1A). 2DE was performed at pH 4–7 IpG strip from 21 days old hydroponically grown rice root tissue in three replicates and images were analyzed by the PDQuest software version 8.0.1 as described above (Figure 1B). The mean value of the high-quality spots was used as the spot quantity on the master gel (Figure 1C, Table S2). For comparative proteomics study minimum 2.5 fold change in protein expression either increase or decrease at any one of the stages was considered for differential spot identification. A criterion of *p* < 0.01 was used to determine the significant difference for analyzing the parallel spots between genotype with analysis of one-way variance (ANOVA). A total of 125 differentially expressed drought responsive protein spots from all time points were subjected to MALDI-TOF/TOF analysis. Out of which 78 spots were identified significantly as indicated by arrows on the higher level matchset image generated by PD Quest (Figure 2) and summarized in Table S3. Some typical gel regions representing protein spots with altered expression are enlarged and shown in Figure 3. These protein spots actually account for 45 distinct proteins (Table 1), suggesting 57% unique protein identifications, while the remaining 43% of the identified differentially expressed proteins either correspond to post-translationally modified forms or may be members of multigene families. Of the 78 identified differentially expressed proteins, 30 protein spots were clearly up-regulated, and 26 were down-regulated, while 22 of the protein spots showed a mixed pattern of development stage dependent expression.

**Table 1:** **List of differentially expressed rice root proteins identified by MS/MS**.

**S. No**.	**Spot ID^a^**	**Protein name**	**GI no**.	**Protein score^b^**	**No. of peptides**	**% coverage**	**Expression pattern^c^**	**Experimental kDa/PI**	**Theoritical kDa/PI**
**BIOENERGY AND METABOLISM (BEM)**									
1.	OsC-3417	Pyruvate dehydrogenase E1 component subunit beta-2, mitochondrial	GI:115480067	48	2	7	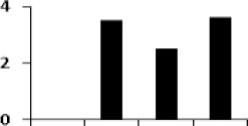	40.3/5.3	64.4/5.3
2.	OsC-4803	Pyruvate dehydrogenase E1 component subunit beta-2, mitochondrial-like [*Setaria italica*]	GI:115434904	100	9	12	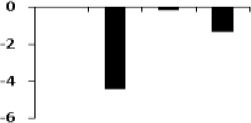	93.9/5.1	97.6/5.5
3.	OsC-2107	Lecithin:cholesterolacyltransferase family protein, expressed [*Oryza sativa* Japonica Group]	GI:218194989	41	1	3	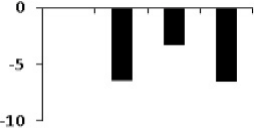	33.6/9.4	29.1/5.2
4.	OsC-3113	ATP synthase subunit d, mitochondrial-like isoform X2 [*Setaria italica*]	GI:115476908	58	3	20	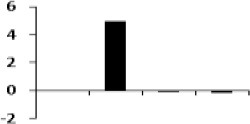	19.7/6.2	9.0/5.4
5.	OsC-5107	Malate dehydrogenase, mitochondrial-like [*Oryza brachyantha*]	GI:115456241	56	2	7	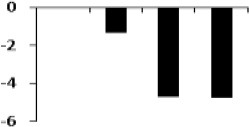	15.9/3.2	29.2/5.8
6.	OsC-7524	Malate dehydrogenase, mitochondrial-like [*Oryza brachyantha*]	GI:115438875	394	6	20	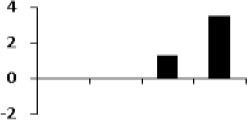	35.6/8.7	76.5/6.3
7.	OsC-7711	Sucrose synthase 1-like isoform X2 [*Oryza brachyantha*]	GI:115453437	170	6	7	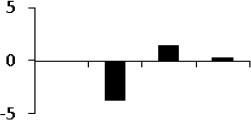	93.3/5.9	90.6/6.4
8.	OsC-3218	Succinyl-CoA ligase [ADP-forming] subunit beta, mitochondrial-like [*Oryza brachyantha*]	GI:115447367	363	11	26	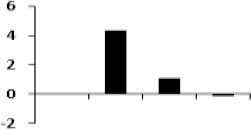	45.4/5.9	47.7/5.5
9.	OsC-8316	Putative acetyl-CoA synthetase [*Oryza sativa* Japonica Group]	GI:49388286	95	4	6	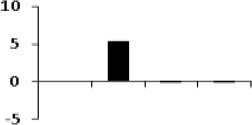	78.5/5.6	57.6/6.4
10.	OsC-1007	Succinate dehydrogenase [ubiquinone] flavoprotein subunit, mitochondrial; [*Oryza sativa* Japonica Group]	GI:75135397	59	2	3	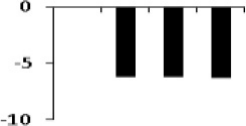	69.4/6.6	28.4/4.9
11.	OsC-8616	Putative succinate dehydrogenase flavoprotein alpha subunit [*Oryza sativa* Japonica Group]	GI:34394418	49	2	3	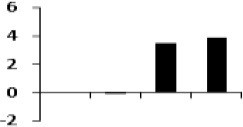	69.4/6.6	86.3/6.6
12.	OsC-2617	Probable nucleoredoxin 1-1; Short, OsNrx1-1 [*Oryza sativa* Japonica Group]	GI:115453457	56	1	2	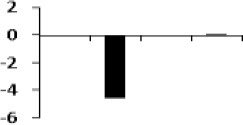	64.0/4.9	80.4/5.1
13.	OsC-3314	Full, Fructokinase-2; [*Oryza sativa* Japonica Group]	GI:115474481	90	3	9	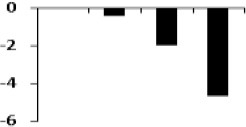	35.8/5.0	54.8/5.4
14.	OsC-3409	Putative transaldolase [*Oryza sativa* Japonica Group]	GI:115441963	72	3	7	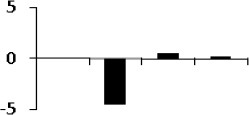	46.5/6.0	69.5/5.4
15.	OsC-3618	V-type proton ATPase subunit B 1-like [*Oryza brachyantha*]	GI:115468606	236	14	31	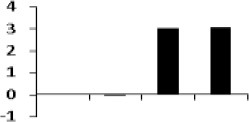	54.1/5.0	79.4/5.3
16.	OsC-4302	Spermidine synthase 1 [*Oryza sativa* Japonica Group]	GI:6468656	76	2	9	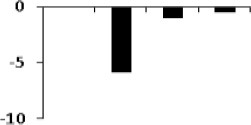	27.9/5.1	50.3/5.5
17.	OsC-5418	Photosystem II subunit D1, partial (chloroplast) [*Polyscias fruticosa*]	GI:345105629	46	2	100	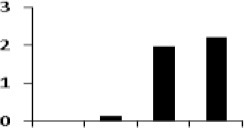	30.9/5.11	62.2/5.9
18.	OsC-7103	Photosystem II subunit D1, partial (chloroplast)	GI:345105629	45	2	100	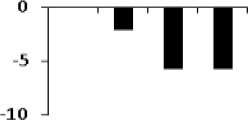	17.2/5.1	28.9/6.1
19.	OsC-5504	Enolase	GI:90110845	737	11	42	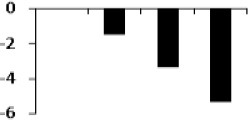	48.2/5.4	70.3/5.7
20.	OsC-7510	Pyrophosphate–fructose 6-phosphate 1-phosphotransferase subunit beta-like [*Oryza brachyantha*]	GI:115467370	180	5	10	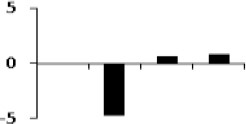	61.9/6.0	78.0/6.4
21.	OsC-8108	Carbonic anhydrase [*Oryza sativa*]	GI:3345477	44	2	10	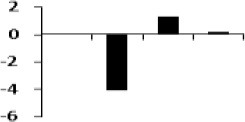	29.4/8.4	35.4/6.8
22.	OsC-8412	Glutamate dehydrogenase 2 [*Oryza sativa* Japonica Group]	GI:81686712	265	4	12	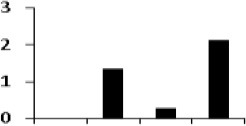	44.9/6.3	62.0/6.8
**CELL DEFENSE AND RESCUE (CDR)**									
23.	OsC-3416	TPA: class III peroxidase 72 precursor	GI:55701011	49	2	6	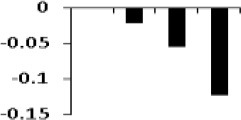	37.8/5.5	59.6/5.3
24.	OsC-4416	TPA: class III peroxidase 72 precursor	GI:55701011	42	2	6	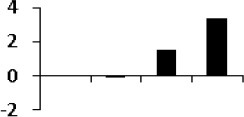	37.8/5.5	66.5/5.5
25.	OsC-6412	TPA: class III peroxidase 72 precursor [*Oryza sativa* Japonica Group]	GI:55701011	60	3	11	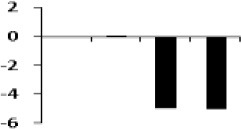	37.8/5.5	67.2/5.9
26.	OsC-8506	TPA: class III peroxidase 72 precursor	GI:38426301	71	4	12	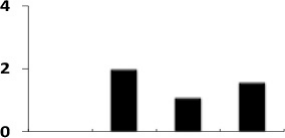	51.7/6.5	71.1/6.5
27.	OsC-3703	Heat shock protein 90	GI:39104468	76	2	3	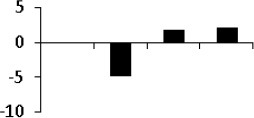	80.4/4.9	89.1/5.2
28.	OsC-8607	Heat shock protein STI-like [*Brachypodium distachyon*]	GI:115447567	140	5	10	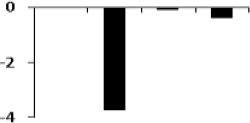	65.1/6.0	84.3/6.6
29.	OsC-4112	24.1 kDa heat shock protein, mitochondrial-like [*Oryza brachyantha*]	GI:115448791	61	3	10	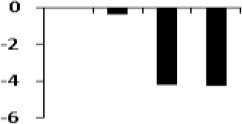	24.0/6.8	29.5/5.7
30.	OsC-4315	Glyoxalase I [*Oryza sativa* Japonica Group]	GI:115475151	185	10	36	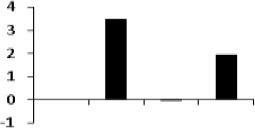	35.1/5.5	55.1/5.6
31.	OsC-5409	Cytosolic monodehydroascorbatereductase [*Oryza sativa* Japonica Group]	GI:4666287	364	5	17	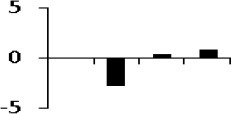	46.7/5.6	61.6/5.7
32.	OsC-7111	GSH-dependent dehydroascorbatereductase 1 [*Oryza sativa* Japonica Group]	GI:6939839	69	1	7	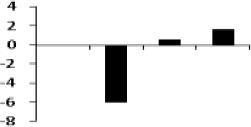	23.7/5.65	35.8/6.3
33.	OsC-3418	Monodehydroascorbatereductase [*Oryza sativa* Japonica Group]	GI:42407947	352	7	24	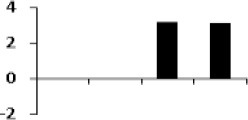	46.7/5.3	65.2/5.3
34.	OsC-7314	Putative r40c1 protein—rice [*Oryza sativa* Japonica Group]	GI:115452789	350	8	27	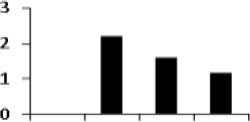	42.2/6.2	57.1/6.4
35.	OsC-7611	Putative stress-induced protein sti1 [*Oryza sativa* Japonica Group]	GI:49388654	76	2	4	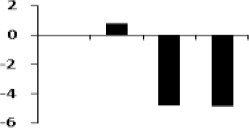	65.1/6.0	84.4/6.3
36.	OsC-7617	Putative stress-induced protein sti1 [*Oryza sativa* Japonica Group]	GI:49388654	126	2	4	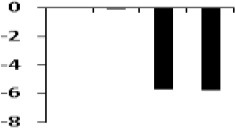	65.1/6.0	85.8/6.4
37.	OsC-8601	Putative stress-induced protein sti1 [*Oryza sativa* Japonica Group]	GI:115447567	90	3	6	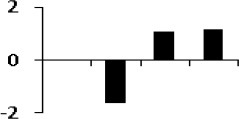	65.1/6.0	84.2/6.5
38.	OsC-2313	Pathogenesis-related protein 1-like [*Oryza brachyantha*]	GI:115474481	472	8	52	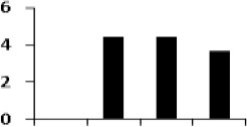	35.8/5.0	53.7/5.0
39.	OsC-2212	Root specific pathogenesis-related protein 10 [*Oryza sativa* Japonica Group]	GI:38678114	179	8	41	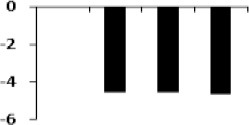	17.0/4.8	41.9/4.9
**PROTEIN BIOGENESIS AND STORAGE (PBS)**									
40.	OsC-4607	Tricin synthase 1-like [*Oryza brachyantha*]	GI:115477092	46	2	10	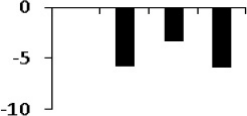	27.9/5.1	84.4/5.6
41.	OsC-1208	Ribosome inactivating protein, expressed [*Oryza sativa* Japonica Group]	GI:29150390	36	1	2	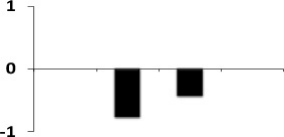	55.6/9.7	46.2/4.9
42.	OsC-1211	Elongation factor 1 beta' [*Oryza sativa* Japonica Group]	GI:218161	74	2	9	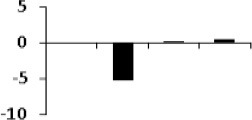	23.8/4.8	44.9/4.9
43.	OsC-7712	Putative elongation factor 2 [*Oryza sativa* Japonica Group]	GI:49387779	58	4	5	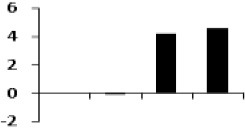	94.9/5.8	94.6/6.4
44.	OsC-2220	Regulation of nuclear pre-mRNA domain-containing protein 1B-like isoform X2 [*Oryza brachyantha*]	GI:20160712	41	1	1	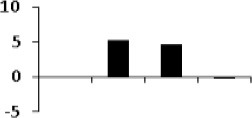	61.2/52.3	48.6/5.0
45.	OsC-3111	Proteasome subunit beta type 3	GI:49388033	86	3	15	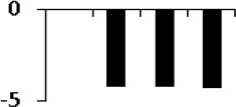	23.1/5.1	35.7/5.4
46.	OsC-5205	Proteasome subunit alpha type-1-like [*Setaria italica*]	GI:115444057	184	10	47	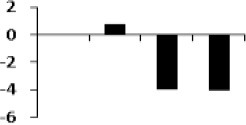	29.8/5.3	47.8/5.7
47.	OsC-3516	Alpha-tubulin	GI:1136120	79	1	3	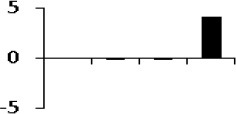	50.4/4.8	69.5/5.4
48.	OsC-4202	Putativetriosephosphateisomerase, chloroplast precursor [*Oryza sativa* Japonica Group]	GI:50725810	126	3	13	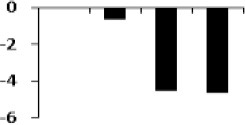	32.7/6.9	39.6/5.5
49.	OsC-4207	Cysteine synthase;	GI:84028195	720	12	48	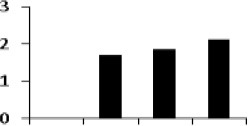	33.9/5.3	47.6/5.6
50.	OsC-4815	Ubiquitin-activating enzyme E1 2, putative, expressed [*Oryza sativa* Japonica Group]	GI:108862075	45	2	1	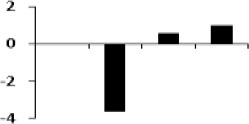	109.3/5.2	97.5/5.5
51.	OsC-7609	5-methyltetrahydropteroyltriglutamate-homocysteine methyltransferase, putative, expressed	GI:108862990	95	4	8	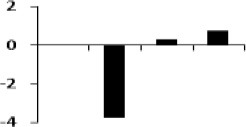	79.2/7.1	87.9/6.3
52.	OsC-7614	5-methyltetrahydropteroyltriglutamate-homocysteine methyltransferase, putative, expressed	GI:108862990	299	7	15	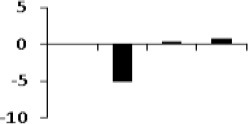	84.6/6.0	88.8/6.4
53.	OsC-8703	5-methyltetrahydropteroyltriglutamate-homocysteine methyltransferase, putative, expressed	GI:108862990	91	4	9	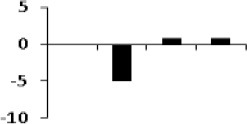	48.2/7.1	88.9/6.5
54.	OsC-3318	Putative thiamine biosynthesis protein	GI:27261025	105	4	16	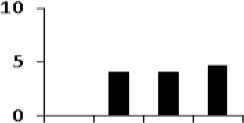	37.1/5.4	52.3/5.3
55.	OsC-7409	Putative beta-alanine synthases [*Oryza sativa* Japonica Group]	GI:22775671	54	2	5	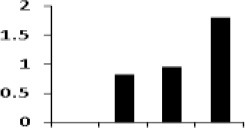	46.0/6.0	62.5/6.4
**SIGNALING (S)**									
56.	OsC-2218	Actin	GI:148886771	327	11	36	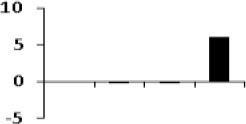	42.1/5.2	41.1/5.1
57.	OsC-2221	Actin-1-like isoform X2 [*Setaria italic*]	GI:115454971	638	9	33	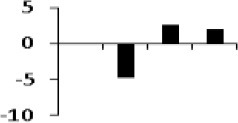	41.8/5.2	46.5/5.0
58.	OsC-5105	Actin-depolymerizing factor 4	GI:75243284	68	5	27	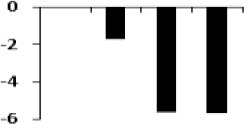	16.0/5.7	29.5/5.8
59.	OsC-2214	Actin-depolymerizing factor 3 [*Zea mays*]	GI:115489014	93	4	23	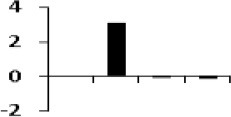	17.0/4.8	46.5/5.0
60.	OsC-2201	14-3-3-like protein GF14-C; AltName: G-box factor 14-3-3 homolog C	GI:115476520	83	2	11	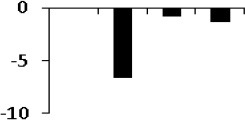	28.9/4.7	44.2/5.0
61.	OsC-4203	Putative membrane protein	GI:9998903	120	4	13	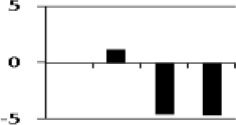	31.5/5.0	47.0/5.5
62.	OsC-5711	Prolylendopeptidase-like [*Oryza brachyantha*]	GI:115470116	67	4	6	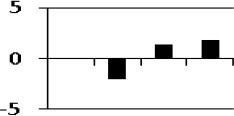	91.5/5.7	89.8/5.8
**UNKNOWN (UK)**									
63.	OsC-1215	Oryzain alpha chain; Flags: Precursor [*Oryza sativa* Japonica Group]	GI:38345906	74	2	5	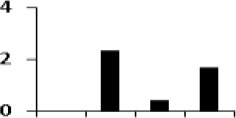	51.3/5.0	48.0/4.9
64.	OsC-2516	Sgt1 [*Oryza sativa*]	GI:6581058	262	4	14	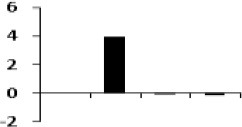	41.0/4.9	71.5/5.1
65.	OsC-3115	Putative probable submergence induced, nickel-binding protein 2A	GI:49388033	53	3	9	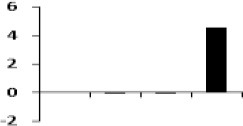	29.7/5.1	36.5/5.3
66.	OsC-3312	Hypothetical protein OsI_37864	GI:125536157	45	2	6	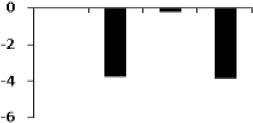	28.9/5.0	56.1/5.4
67.	OsC-3413	Hypothetical protein	GI:54290369	45	1	9	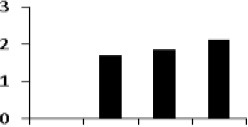	17.2/9.2	59.8/5.4
68.	OsC-3509	3'-N-debenzoyl-2'-deoxytaxol N-benzoyltransferase-like [*Oryza brachyantha*]	GI:115438576	129	3	8	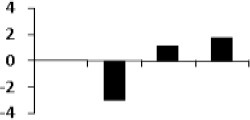	46.1/5.1	69.9/5.4
69.	OsC-3518	Hypothetical protein OsI_15081	GI:218194450	57	2	1	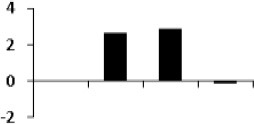	214.5/7.8	71.7/5.4
70.	OsC-4107	Hypothetical protein OsJ_00565 [*Oryza sativa* Japonica Group]	GI:125569214	155	3	23	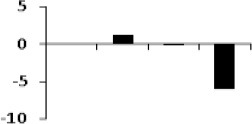	17.9/5.8	28.9/5.5
71.	OsC-4213	Unknown protein [*Oryza sativa* Japonica Group]	GI:30017570	60	3	10	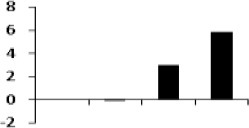	35.7/9.6	41.9/5.7
72.	OsC-4510	Hypothetical protein OsI_31140	GI:125563499	148	5	12	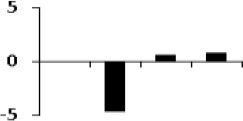	51.0/5.7	70.4/5.6
73.	OsC-4808	Hypothetical protein OsI_02498	GI:218188499	53	2	2	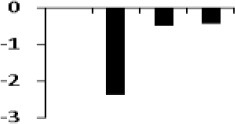	96.9/5.5	102.3/5.6
74.	OsC-6105	Hypothetical protein OsJ_22407 [*Oryza sativa* Japonica Group]	GI:222636106	45	1	3	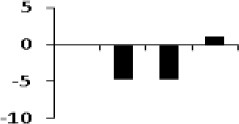	30.5/7.4	36.2/6.0
75.	OsC-6201	Hypothetical protein OsJ_04535	GI:125573095	46	1	8	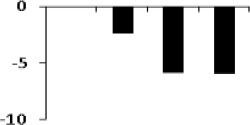	29.6/10.3	43.2/5.8
76.	OsC-6607	PREDICTED: uncharacterized protein LOC100818188 isoform X1 [*Glycine max*]	GI:571541740	45	7	15	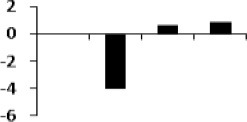	43.6/5.4	82.9/5.9
77.	OsC-8408	Hypothetical protein OsI_23019	GI:218198209	118	7	23	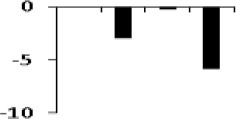	41.3/6.6	60.9/6.7
78.	OsC-8717	Hypothetical protein SORBIDRAFT_03g034200	GI:115456914	806	13	21	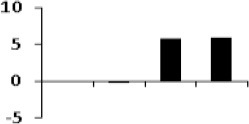	94.9/5.8	95.6/6.5

a Spot number as given on the two dimensional gel images. The first letters (Os) represent the source plant Oryza sativa followed by the fraction cytoplasm (c).

b The significance score (P < 0.05) of a protein, as produced by the Mascot algorithm.

c Protein expression profile represents the average change in spot density among different stages of drought stress. The data were taken in terms of fold expression with respect to the control value and were log transformed to the base 2 in order to level the scale of expression and to reduce the noise.

### Functional distribution of dehydration responsive proteins

**Figure 4:**
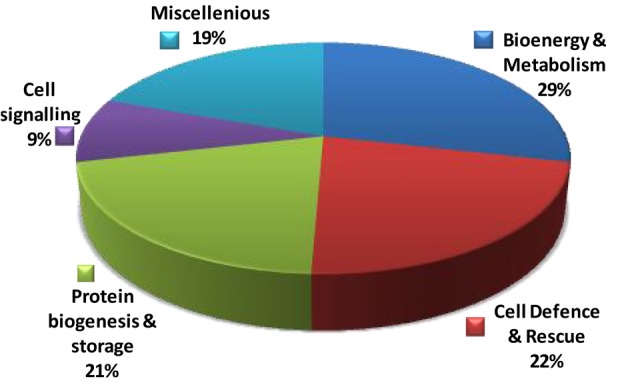
**Distribution of 78 identified differentially expressed rice root proteins in five functional classes based on their putative functions assigned them using protein database**.

To understand the function of the proteins associated with drought induced changes in the rice roots, the differentially expressed proteins were sorted into different functional categories. Seventy-eight identified differentially expressed proteins could be assigned to five functional classes based on their putative roles in the drought-response (Figure 4, Table 1). In case of Heena the largest percentage of the identified proteins was involved in bioenergy and metabolism (29%), cell defense and rescue (22%), protein biogenesis and storage (21%), cell signaling 9%, and miscellaneous (19%; Figure 4). In a number of cases, many proteins were represented by multiple isoelectric forms (Table 1), suggesting the possible post-translational modification of the candidate protein.

### Bioenergy and metabolism (BEM)

The BEM class contained 24 proteins which appeared as differential spots. ATP synthase subunit d (OsC-3113), malate dehydrogenase, mitochondrial-like (OsC-5107), succinyl-CoA ligase [ADP-forming] subunit beta (OsC-3218), putative acetyl-CoA synthetase (OsC-8316), V-type proton ATPase subunit B 1-like (OsC-3618), glutamate dehydrogenase 2 (OsC-8412), putative succinate dehydrogenase flavoprotein alpha subunit (OsC-8616) were upregulated, while pyruvate dehydrogenase E1 component subunit beta-2 (OsC-3417), malate dehydrogenase, mitochondrial-like (OsC-7524), succinate dehydrogenase [ubiquinone] flavoprotein subunit, mitochondrial (OsC-1007), probable nucleoredoxin (OsC-2617), fructokinase-2 (OsC-3314), spermidine synthase 1(OsC-4302), pyruvate dehydrogenase E1 component (OsC-4803), lecithin:cholesterolacyl transferase family protein (OsC-2107), and enolase (OsC-5504) were downregulated. Other proteins like sucrose synthase 1-like isoform X2 (OsC-7711), putative transaldolase (OsC-3409), pyrophosphate-fructose 6-phosphate 1-phosphotransferase (OsC-7510) and carbonic anhydrase (OsC-8108) showed mixed pattern of expression.

Alteration in bioenergy metabolism under drought can be addressed by increase in abundance of enzymes involved in acetyl CoA synthesis, TCA cycle, transport proteins, synthesis of osmoprotectants, and protein metabolism. Acetyl-CoA synthetase (OsC-8316) showed a 43 fold increase in abundance on first day and 66 fold on the third day of drought induction (Table 1); however the expression remains similar as control on the 7th day. The enzyme catalyzes the formation of acetyl CoA from acetate utilizing inorganic phosphate (Lin and Oliver, 2008) and can be employed in the TCA cycle during aerobic respiration for energy production and electron carriers. It is to be noted here that drought stress resulted in downregulation of pyruvate dehydrogenase (OsC-3417), hence the upregulation of Acetyl-CoA synthetase possibly provided an alternate source of energy production (Schwer et al., 2006).

The TCA cycle enzymes like succinyl co-A ligase (OsC-3218), succinate dehydrogenase (OsC-1007) and malate dehydrogenase (OsC-7524) were upregulated. Succinyl-CoA ligase catalyzes the reversible inter conversion of succinyl-CoA to succinate (Cavalcanti et al., 2014). There was a 20 fold increase in the enzyme abundance at first day (Table 1). The result corroborates with earlier studies which reported increase in enzyme expression in wheat roots in response to aluminum stress (Drummond et al., 2001). Succinate dehydrogenase catalyzes the oxidation of succinate to form fumarate (Singer et al., 1973; Jardim-Messeder et al., 2015). The enzyme is an important component of TCA cycle as well as the electron transport chain (Popova et al., 2010; Huang and Millar, 2013). Two isozymes of succinate dehydrogenase were found as differential spots, of which one (OsC-1007) was downregulated while the other (OsC-8616) was upregulated showing 28 fold increase in activity on the third day (Table 1). The transcription of succinate dehydrogenase flavoprotein subunit has been found to be up-regulated in *Ilex paraguariensis* leaves in response to water deficit and ABA application (Acevedo et al., 2013). Furthermore, its increased activity results in root elongation (Huang et al., 2012), and also has a role in plant adaptation toward stress and especially by combating reactive oxygen species (Pastore et al., 2007). Of the two isozymes of malate dehydrogenase identified, one was downregulated (OsC-5107), while the other isozyme (OsC-7524) showed 5 and 11 fold increase in activity on third and seventh day of drought stress, respectively (Table 1). ATPases are integral transport proteins that help in the hydrolysis of ATP as well as movement of protons across membranes to generate electrochemical gradients (Palmgren and Harper, 1999). The action of ATPase can influence stress mechanism by changing the membrane potential and proton gradient (Elmore and Coaker, 2011). Transcript and protein levels of ATPase have been reported to increase under salt stress conditions (Batelli et al., 2007). Interestingly V-type proton ATPase subunit B 1-like protein (OsC-3618) and ATP synthase subunit d mitochondrial-like isoform X2 (OsC-3113) were upregulated. While, approximately 8 fold increases in activity of V-type proton ATPase was noted on third and seventh day; the activity of mitochondrial ATP synthase increased to approximately 33 fold in first day and 58 fold in third day (Table 1).

Mitochondrial pyruvate dehydrogenase (OsC-4803) is an intermediate enzyme that links glycolysis to the citric acid cycle. The enzyme was downregulated under drought stress and results substantiate the earlier reports (Simova-Stoilova et al., 2015). Fructokinase-2 (OsC-3314) is mainly involved in carbohydrate metabolism, more specifically, sucrose and fructose metabolism (Odanaka et al., 2002). Previously rice fructokinase showed elevated levels of expression during infection with the fungus, *Magnapor theoryzae* (Ryu et al., 2006). Sucrose synthase (OsC-7711), a major protein of energy metabolism, plays an important role in controlling the mobilization of sucrose into various pathways essential for various metabolic, structural, and other storage functions of the plant cell (Hesse and Willmitzer, 1996). The protein expression followed a miscellaneous pattern with 2.7 fold abundance on third day (Table 1), indicating an alteration in enzyme function more toward osmotic adjustment and storage compounds (Hasibeder et al., 2015).

### Cell defense and rescue (CDR)

Seventeen differentially expressed proteins related to cell defense and rescue were identified, out of which pathogenesis-related protein 1 (PR1, OsC-2313), one isoform of TPA: class III peroxidase 72 precursor (OsC-4416), glyoxalase I (OsC-4315), putative r40c1 protein (OsC-7314), stress-induced protein sti1 (OsC-7617, OsC-8601), and monodehydroascorbate reductase (OsC-3418) were upregulated whereas root specific pathogenesis-related protein 10 (OsC-2212), 24.1 kDa heat shock protein (OsC-4112), isozyme of monodehydroascorbate reductase (OsC-5409), TPA: class III peroxidase 72 precursor (OsC-6412 and OsC-3416), isozyme of stress-induced protein sti1 (OsC-7611) and heat shock protein STI (OsC-8607) were downregulated, while heat shock protein (HSP)-90 (OsC-3703) and GSH-dependent dehydroascorbatereductase 1 (OsC-7111) showed miscellaneous pattern of expression.

The ROS scavengers provide cells with an efficient machinery for detoxifying ${\text{O}}_{2}^{•-}$ and H_2_O_2_ and constitute the first line of defense. A considerable upregulation of these enzymes was a major tolerance mechanism adopted by the variety. The plant class III peroxidases are known to basically involved in metabolism of ROS along with some other function like auxin metabolism, lignin and suberin formation and cross-linking of cell wall components (Almagro et al., 2009). The class III peroxidase (OsC-4416) expression gradually increased up to 11 fold from first to seventh day (Table 1). Since the protein is involved in lignin biosynthesis, a gradual increase in expression suggests enhanced lignin production. Increased amount of lignin builds the mechanical strength of cell wall and thereby protects roots against the dry soil. In addition, cell wall modification is also used to minimize water loss and cell dehydration, thus helping plants to resist and recover from drought upon availability of water (Yoshimura et al., 2008). An 11 fold increase in expression was observed in glyoxalase 1 (OsC-4315), which is a component of the glyoxalase system that carries out the detoxification of methylglyoxal and the other reactive aldehydes produced as a normal part of metabolism (Vander, 1989). Glyoxalase 1 has been identified as a salt induced proteome in rice roots and plays an important role in methylglyoxal detoxification in salt tolerant rice, thus ensuring redox homeostasis (El-Shabrawi et al., 2010). Putative r40c1 protein showed increased abundance in response to drought. The exact function of the protein is unknown; however its upregulation has been correlated to drought tolerance (Kumar et al., 2015). The expression of monodehydroascorbate reductase (OsC-5409) was increased by 9 fold on seventh day. It is a critical component of the ROS scavenging system in rice, and its expression can improve drought tolerance in rice (Wang et al., 2005). Interestingly, the expression of pathogenesis related protein-1 (PR1) was 22 fold increased within 24 h of drought induction; however root specific pathogenesis related protein-10 (PR10) was down regulated in response to drought. PR1 gene expression is salicylic-acid responsive and induced in response to a variety of pathogens (Elvira et al., 2008) while PR-10 protein expression is induced through activation of jasmonic acid signaling pathway (Hashimoto et al., 2004). Thus, it might be suggested that drought stress might responsible in increase expression of SA regulated PR proteins and decreases in JA-regulated defense.

### Protein biogenesis and storage (PBS)

A total of 15 proteins were found as differential spots, out of which nuclear pre-mRNA domain-containing protein 1B-like isoform X2 (OsC-2220), alpha-tubulin (OsC-3516), putative elongation factor 2 (OsC-7712), putative thiamine biosynthesis protein (OsC-3318), putative beta-alanine synthase (OsC-7409), and cysteine synthase (OsC-4207, OsC-4207) was upregulated; tricin synthase 1 (OsC-4607), putative triose phosphate isomerase (OsC-4202), proteasome subunit alpha type-1 (OsC-5205) was downregulated; while three isozymes of 5-methyl tetra hydropteroyl triglutamate-homocysteine methyltransferase (OsC-7609, OsC-7614, OsC-8703), elongation factor 1 beta (OsC-1211), proteasome subunit beta type 3 (OsC-3111) and ubiquitin-activating enzyme E1 2, putative (OsC-4815) showed miscellaneous pattern of expression.

Cysteine synthase is a key enzyme for mediating abiotic tolerance owing to the production of antioxidants and metal chelators, such as glutathione, metallothionein, and phytochelatin. In an earlier study cysteine synthase was found to be increased in the roots of wheat during stress (Yang et al., 2007). We also observed significantly upregulation of cysteine synthase (OsC-4207) in tolerant variety used for analysis. Tubulin class of proteins is known to play a critical role in cell division and elongation and alpha-tubulin (OsC-3516) has been specifically reported in roots of drought tolerant rice verities (Rabello et al., 2008). Elongation factor 2 (OsC-7712) which showed 24 fold increases in abundance is reportedly a stress responsive protein which is mostly downregulated in sensitive verities of rice (Chen et al., 2015). There was a decrease in abundance of proteasome subunit beta type 3 protein (OsC-5205) in response to drought while ubiquitin activating enzyme (OsC-4815), required for ubiquitination, showed a gradual increase in abundance and increased 2-fold by the end of seventh day (Table 1). The protein ubiquitination plays a central role in regulating the transcriptional changes required for adaption and survival strategies of plants to different environmental stresses (Dametto et al., 2015). Glutamate dehydrogenase expedites amino acid synthesis in drought stress conditions where nitrogen assimilation is decreased and also known to maintain homeostasis during stress (Masclaux-Daubresse et al., 2010). Present study showed a 4 fold increase in glutamate dehydrogenase (OsC-8412) expression on seventh day (Table 1). Putative thiamine biosynthesis protein (OsC-3318) showed an 18 fold increase on first day and 27 fold increase on the seventh day. Recent reports suggest that vitamin B1 (thiamine) participates in the processes underlying plant adaptations to various types stress conditions including cold, heat, drought, and other oxidative stress (Rapala-Kozik et al., 2012). A gradual increase in putative beta-alanine synthase (OsC-7409) suggests significant enrichment of beta-alanine which functions as an osmoprotectant under drought conditions in various plant systems (Ranjan et al., 2012).

### Cell signaling

The response of roots to water limiting conditions seems to be crucial to trigger drought tolerance mechanisms, since roots are one of the primary sites for stress signal perception in which a signaling mechanism initiates a cascade of gene expression responses to drought. These changes in protein profile can result in successful adaptations leading to stress tolerance by regulating protein expression and signal transduction in the stress response (regulatory proteins) or directly protecting the plant against environmental stress (functional proteins). Seven cell signaling related proteins were identified as differential spots, out of which actin (OsC-2218) and actin-1-like isoform X2 (OsC-2221) were significantly upregulated. Whilst a 20-fold increase in expression of actin-depolymerizing factor 3 (OsC-2214) was observed on third day (Table 1), a consistent decrease in the expression of actin-depolymerizing factor 4 (OsC-5105) was noticed during the study. Other proteins like the 14-3-3-like protein (OsC-2201), putative membrane protein (OsC-4203), and prolyl endopeptidase-like protein (OsC-5711) showed miscellaneous pattern of expression in response to drought. Actin depolymerizing factors (ADFs) are small actin-binding proteins and are known to confer abiotic drought tolerance (Huang et al., 2012). There was an increase in abundance of actin depolymerizing factor 3 (OsC-2214) by 8 and 20 fold, respectively, on first and third day of drought induction, however the expression came down as similar to control at seventh day, thus suggesting its regulatory role in drought tolerance. During signal transduction, 14-3-3 proteins mediate several protein-protein interactions through post-translational modification like phosphorylation which ultimately affects multiple plant functions (Chen et al., 2006). Surprisingly, a non-coherent expression of 14-3-3 protein (OsC-2201) was observed under drought stress. A decrease in prolyl endopeptidase (OsC-5711) expression was observed on first of drought induction; however an increase in its abundance was noted at third day and the abundance increased by 3.7-fold at the end of seventh day.

### Miscellaneous

The proteins with functions other than the defined categories were ascribed as miscellaneous proteins and constituted 22% of the differentially expressed protein. Amongst the differentially expressed miscellaneous proteins, Sgt1 (OsC-2516), submergence induced, nickel-binding protein 2A (OsC-3115), Oryzain alpha chain; Flags: Precursor [*O. sativa* Japonica group] (OsC-1215), hypothetical protein (OsC-3413), hypothetical protein OsI_15081 (OsC-3518), and hypothetical protein SORBIDRAFT_03g034200 (OsC-8717) were upregulated; hypothetical protein OsI_37864 (OsC-3312) was downregulated, while 3′-N-debenzoyl-2′-deoxytaxol N-benzoyltransferase (OsC-3509) and uncharacterized protein LOC100818188 (OsC-6607) showed varied expression.

SGT1 (OsC-2516) is a highly conserved protein among eukaryotes that binds specifically to the molecular chaperone, HSP90 and helps in regulation of resistance extended by many resistance proteins (Azevedo et al., 2006; Wang et al., 2010). It is interesting to note here that there was an upregulation of HSP90 (protein abundance increased to 4.6-fold) with SGT1 in response to drought in Heena roots might involve in drought tolerance. The nickel-binding protein 2A is the member of dehydration-responsive element-binding protein 2A family which acts as a transcriptional activator that binds specifically to the cis-acting dehydration-responsive element (DRE) to regulates high salinity and dehydration-inducible transcription (Dubouzet et al., 2003). A 23-fold increase in protein abundance suggests an important role of nickel-binding protein 2A (OsC-3115) in conferring drought tolerance to Heena, however its exact function as a drought resistant protein needs to be further elucidated. Another hypothetical protein SORBIDRAFT_03g034200 (OsC-8717) showed a 63-fold increase in abundance on seventh day which indicates its important role in conferring drought tolerance in later stage, however it needs further elucidation.

### Cluster analysis of drought responsive protein

**Figure 5:**
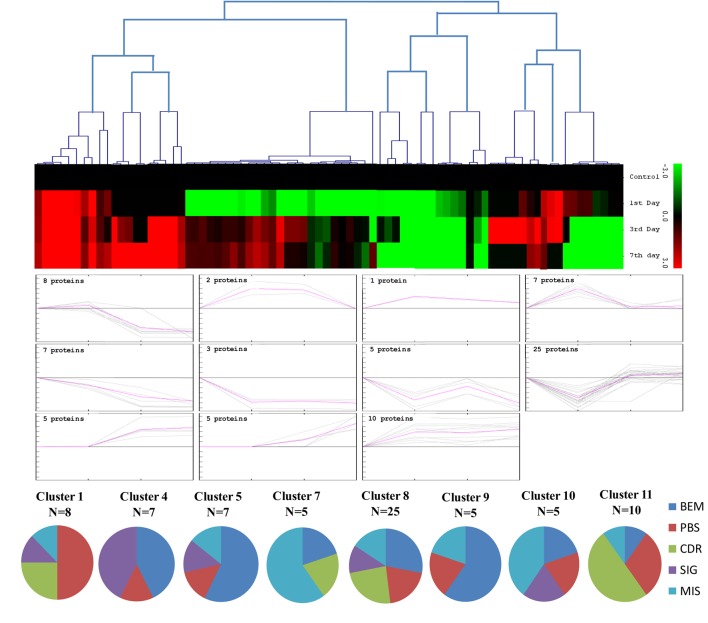
**Expression clustering of 78 differentially expressed proteins showing 8 clusters based on their expression profiles**. The gray lines represent expression profile of protein separately and the mean expression profile is indicated by pink line. Total number of proteins with same expression profile is provided in inset within the respective cluster. Detailed information on proteins within each cluster present in Figure S1.

The unbiased hierarchical clustering method, SOTA was used to study the correlated expression pattern of the drought responsive proteins. This method allows integration of the multiple proteins showing similar expression profiles which provide comprehensive overview of the rice root protein network regulation in coordination. The data were log transformed to the base of 2 to reduce the noise and level the scale of fold-expression for SOTA clustering analysis. The analysis grouped 78 proteins into 11 distinct clusters (Figure 5), allowing a maximum diversity of 0.8 within a single cluster. Figure S1 represents the detailed information on proteins within each cluster. Only those clusters which is having *n* ≥ 5 (n represents number of identified proteins) were considered for coordinated expression profile of the drought responsive proteins. Analysis revealed that cluster 8 contains maximum number of proteins with similar expression followed by cluster 11. The proteins of cluster 8, displayed decreased accumulation during initial stage of drought and subsequent increase at late stages. While in cluster 1, maximum number of the proteins displayed increased accumulation during initial stage of drought and then decrease at late stage. Most of the clusters except cluster 8 showed selective representation for specific functional classes of proteins. The proteins in cluster 9, 10, and 11 were found to be over-expressed throughout drought stress in one or all stages, whereas in cluster 5 and 7 showed only down-regulated proteins. The cluster 4 which contain 7 proteins were expressed immediately in day 1 then disappear throughout suggested their role in helping in plant to tolerate drought shock. The drought responsive proteins with miscellaneous function were found to be distributed in almost all the clusters. SOTA clustering analysis may provide useful information for their putative function in drought tolerance based on the abundance in rice root.

#### Validation of proteomics data of selected proteins with qRT PCR

**Figure 6:**
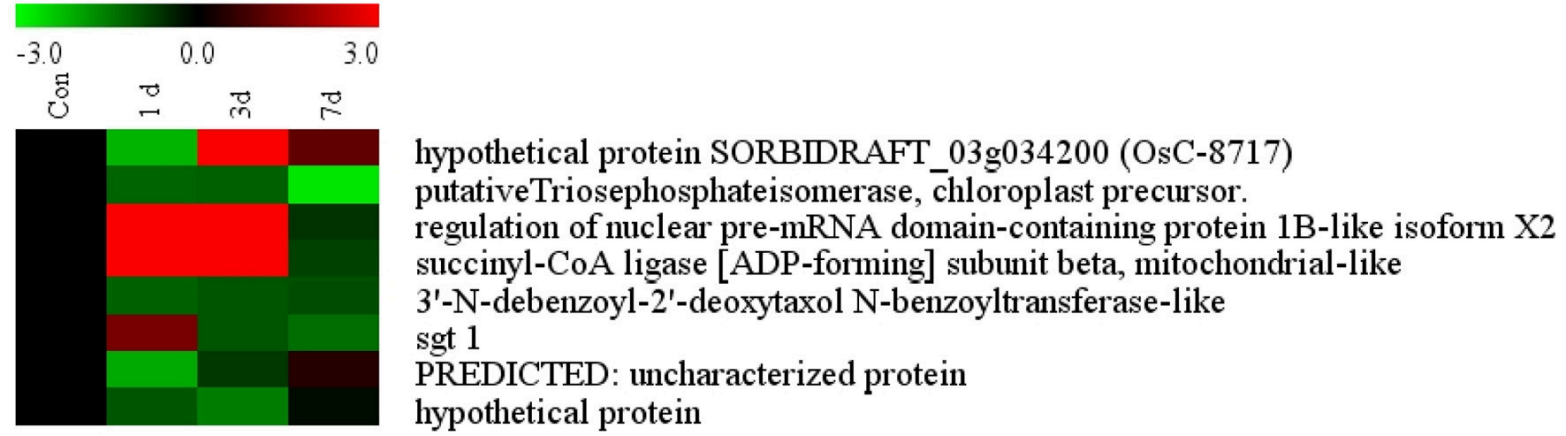
**Quantitative validation of relative expression of selected transcripts related to different functional classes of rice root**. Heat map of some differentially regulated known and hypothetical protein showing their relative expression by qRT-PCR along with their protein expression data obtained from 2DE. The signal intensity of each transcript was normalized using ubiquitin house keeping gene. The top most bar represents the fold change expression values of selected genes.

The list of putatively regulated proteins depicted in Table 1 is a snapshot of proteins from rice root. We have selected 5 genes with miscellaneous function for comparing their expression with proteome data. Among them, a highly conserved eukaryotic protein, SGT1 (OsC-2516) shows its binding specifically to the molecular chaperone to regulation of resistance extended by many resistance proteins (Wang et al., 2010). An upregulated TCA cycle enzymes succinyl co-A ligase (OsC-3218) catalyzes the reversible inter conversion of succinyl-CoA to succinate (Cavalcanti et al., 2014). Triose phosphate isomerase (OsC-4202) is required to produce ATP during glycolysis and it has been reported to have highly induced by salt or drought in rice leaves (Lee et al., 2011). Benzoyltransferase (OsC-3509) have role in final step of acylation of taxol biosynthesis pathway but its role in drought stress is hitherto unknown. Apart from these, some other unknown genes like hypothetical protein (OsC-6201), PREDICTED: uncharacterized protein (OsC-6607), hypothetical protein SORBIDRAFT_03g034200 (OsC-8717) were also taken to check their abundance. However, expression profile of these proteins were somewhat similar as determined by the 2-DE analysis (Table 1), nevertheless, there was difference in fold-induction in protein expression (Figure 6, Table 1). Since all the differentially expressed proteins were not identified, it can be assumed that some other isoforms of the same protein might be part of the root proteome. The post-translational modifications of some of the differentially expressed proteins might also affect the actual expression levels determined by two different techniques. Further study may explore such type of correlation in a better way.

## Conclusion

**Figure 7:**
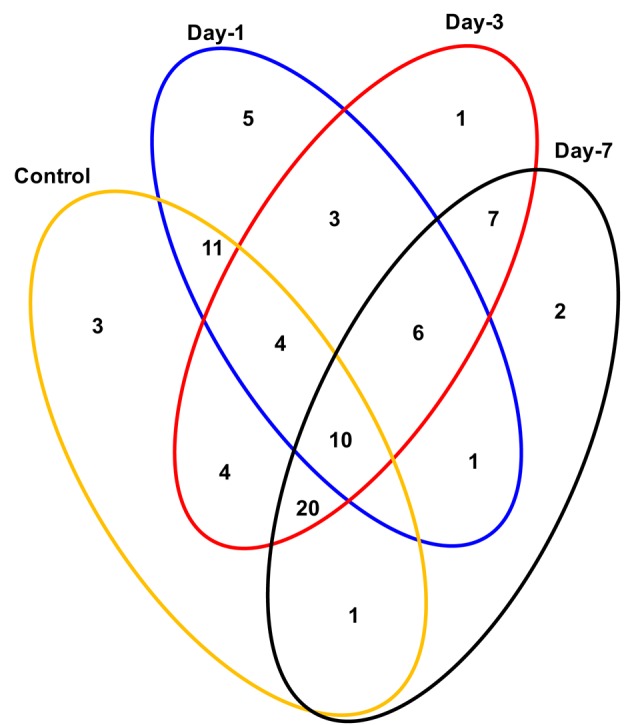
**Venn diagram showing the distribution of differentially expressed proteins in time-specific and overlapping manner during drought stress**. The areas shown in the diagram are not proportional to the number of proteins in the groups.

**Figure 8:**
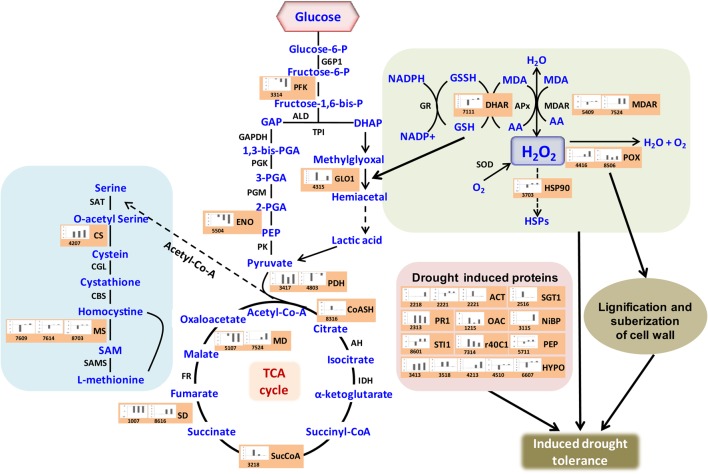
**Illustration of role of differentially regulated proteins involved in different pathways in rice for sustaining during drought stress**. Proteins engaged in carbon breakdown, protein synthesis and ROS pathway are displayed on the corresponding metabolic pathways. Graphs are the representatives of expression profile of individual protein and number given below in each graph indicates the protein identification number.

Crop plants including rice exhibit several adaptive and acclimatization strategies to combat environmental conditions such as drought. Such strategies include from visible phenotypic changes to complex physicochemical traits. At molecular level, these traits, often represented by differentially regulated proteins during stress and may serve as important stress tolerance markers. We report here a systematic proteomic analysis of the rice root proteins under PEG-simulated drought stress conditions. In conclusion, the root-specific comparative proteomes of rice identified a number of proteins that are putatively associated with stage specific drought tolerant. Of the 78 differentially expressed proteins, 10 were found to be differentially regulated in all the four stages during drought stress. Three proteins exclusively expressed in the control, were disappear after drought stresses. Total 8 proteins found to be newly synthesized during early or later stage of drought stress, implying their possible role in drought tolerance (Figure 7). Functional classification revealed that maximum number of proteins fall in the category of bioenergy and metabolism followed by those involved in cell defense (Figure 4). The higher number of metabolic proteins offers a unique opportunity to predict more activity toward carbon assimilation, respiration, and storage products like sucrose and starch to combat drought condition. Based on results from present study, a putative model elucidating the role of the differentially expressed proteins in different pathways like glycolysis, TCA, amino acid metabolism, ROS, and other possible mechanism(s) underlying dehydration tolerance is in Figure 8. A large number of proteins with miscellaneous functions are matter of further investigation for their putative role in drought tolerance. However, the present proteomics study could represent only a small part of the rice proteome, further investigation to assign their putative biological functions may be useful for a better understanding of complex biological traits, such as drought tolerance. Many other drought-responsive proteins still need to be identified with advancement to technology which may help in better understanding of the drought response in rice.

## Author contributions

CN, DC, and PC designed the experiment. LA, SG, and SM performed the experiments. LA, GP, and SK analyze the data and wrote the paper.

## Funding

The study was supported by New Initiative (as a Cross Flow Technology project) “Root Biology and its Correlation to Sustainable Plant Development and Soil Fertility (BSC0204)” from Council of Scientific and Industrial Research (CSIR), New Delhi, India and JC Bose Fellowship, Science and Engineering Research Board, Department of Science and Technology, Government of India, awarded to CN.

### Conflict of interest statement

The authors declare that the research was conducted in the absence of any commercial or financial relationships that could be construed as a potential conflict of interest.

## Supplementary material

Figure S1 **Heat map revealing the expression of differentially regulated proteins of rice root present within each cluster**.

Table S1 **List of primers used for Real Time PCR**.

Table S2 **Reproducibility of 2-D gels**.

Table S3 **List of rice root proteins identified by MS/MS analysis**.

## Abbreviations

SAT: serine acetyl transferase
CS: cystein synthesae
CGL: Cystathionine γ-lyase
CBS: Cystathionine β-synthase
MS: methionine synthase
SAM: S-adenosylmethionine
SAMS: S-adenosylmethionine synthesae
G-6-P: Glucose 6 phosphate
PFK: pyrophosphate-dependent phosphofructokinase
ALD: aldehyde dehydrogenase
TPI: triose phosphate isomerase
GAPDH: glyceraldehyde 3-phosphate dehydrogenase
PGK: phosphoglycerate kinase
PGM: phosphoglycerate mutase
ENO: enolase
PK: pyruvate kinase
GLO1: glyoxalase 1
PDH: Pyruvate dehydrogenase
CoAS: Co-A synthesae
AH: aconitate hydratase
IDH: isocitrate dehydrogenase
SucCoA: Succinyl Co-A synthese
SD: succinate Dehydrogenase
FR: fumarase
MD: malate dehydrogenase
GR: glutathione reductase
DHAR: dehydroascorbate reductase
APx: ascorbate peroxidase
MDAR: monodehydroascorbate reductase
POX: peroxidases
SOD: superoxide dismutase
HSPs: heat shock proteins
ACT: actin
SGT1: suppressor of the G2 allele of *skp*1
PR: pathogenesis related protein
OAC: oryzain alpha chain
NiBP: Ni binding protein
STI1: stress inducible protein
PEP: phosphoenolpyruvate
HYPO: hypothetica.
