# Supplementary material for: Elucidation of Complex Nature of PEG Induced Drought-Stress Response in Rice Root Using Comparative Proteomics Approach
Source: Front Plant Sci. 2016 Sep 29;7:1466. doi: 10.3389/fpls.2016.01466 (PMC5040710; doi:10.3389/fpls.2016.01466)

Supplementary Material

Elucidation of complex nature of PEG induced drought-stress response in rice root using comparative proteomics approach

Lalit Agrawal, Swati Gupta*, Shashank Kumar Mishra*, Garima Pandey, Susheel Kumar, Puneet Singh Chauhan, Debasis Chakrabarty, Chandra Shekhar Nautiyal[#]

[#]Correspondence:

Chandra Shekhar Nautiyal Email: nautiyalnbri@lycos.com, Telephone: +91-522-2205848, Fax: +91-522-2205839

Supplementary Figure S1: Heat map of expression of differentially regulated proteins of rice root present within each cluster.

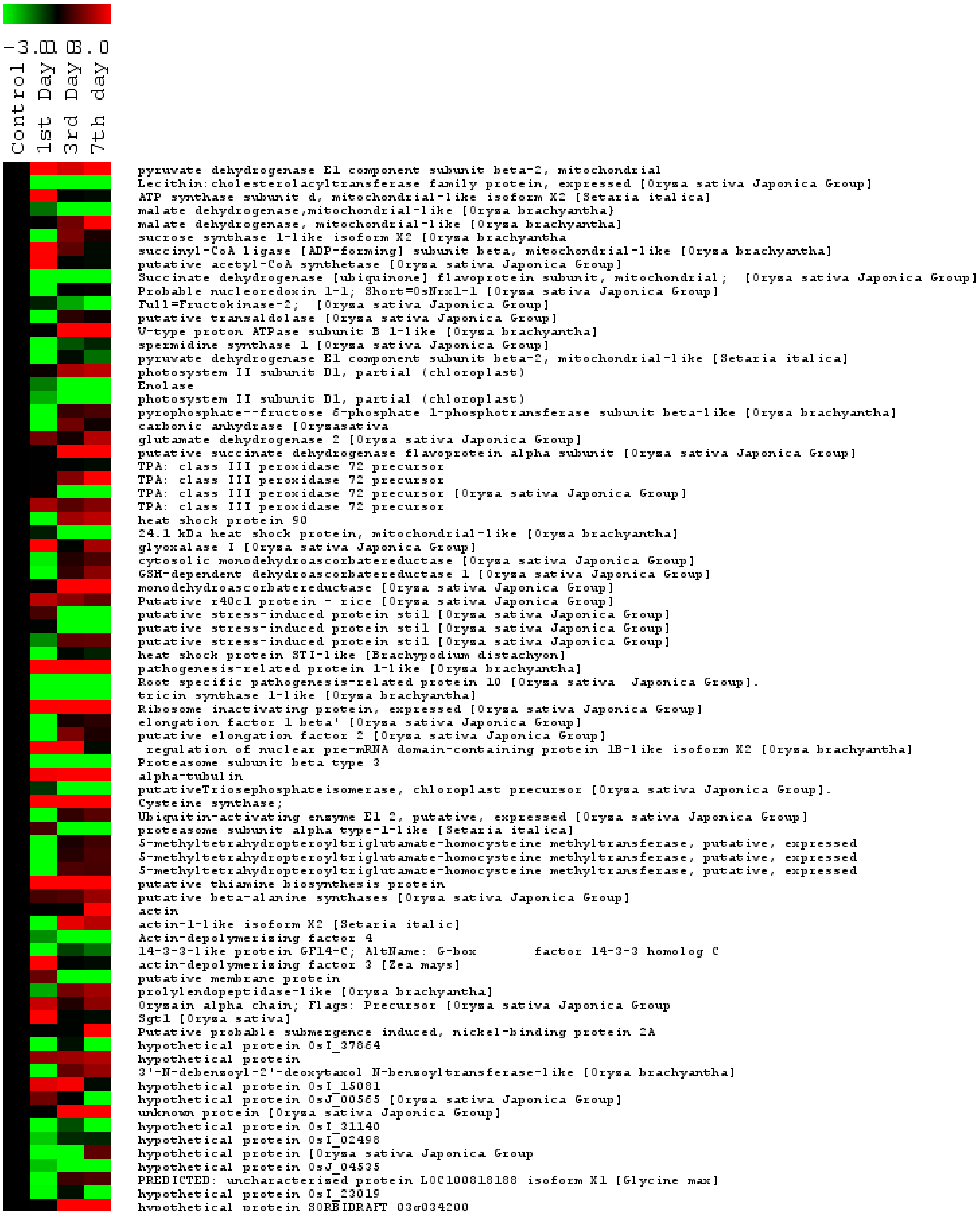

Supplement: Figure S1 — Heat map revealing the expression of differentially regulated proteins of rice root present within each cluster. [file DataSheet3.pdf]
