# Supplementary material for: Elucidation of Complex Nature of PEG Induced Drought-Stress Response in Rice Root Using Comparative Proteomics Approach
Source: Front Plant Sci. 2016 Sep 29;7:1466. doi: 10.3389/fpls.2016.01466 (PMC5040710; doi:10.3389/fpls.2016.01466)
Supplement: Table S1 — List of primers used for Real Time PCR. [file DataSheet1.doc]

***Supplementary Material***

**Elucidation of complex nature of PEG induced drought-stress response in rice root using comparative proteomics approach**

Lalit Agrawal, Swati Gupta*, Shashank Kumar Mishra*, Garima Pandey, Susheel Kumar, Puneet Singh Chauhan, Debasis Chakrabarty, Chandra Shekhar Nautiyal^#^

^#^Correspondence:

Chandra Shekhar Nautiyal Email: nautiyalnbri@lycos.com, Telephone: +91-522-2205848, Fax: +91-522-2205839

**Supplemental Table** S1. List of primer sets used for quantification of selected candidate genes by real time PCR.

| **S. No.** | **Primer name** | **Primer sequence (5’ to 3’)** |
| --- | --- | --- |
| 1 | DD-benzoyltransferase-F | AGGCGCTCGTGCACTACTAC |
| 2 | DD-benzoyltransferase-R | TTGCCCATCAAAGACCAAGT |
| 3 | sti1-F | GCACTTCACTGACGCCATC |
| 4 | sti1-R | CTTCAGCTCGACGGTCTTCT |
| 5 | UNK- F | CGGCACAACAAAAATCAACC |
| 6 | UNK-R | TGATTGCCTGGAGTCAACAT |
| 7 | HYP-F | CGAGCGTGTACCCATTCCT |
| 8 | HYP-R | AGACCGATGATGGTGATGGT |
| 9 | Succinyl-CoA ligase-F | TGATGACAATGCTGCCTTTA |
| 10 | Succinyl-CoA ligase-R | ATTTCTCCATCAAGCCCAAT |
| 11 | Triose phosphate isomerase-F | CTGTTCGTGATTGGTTGAAA |
| 12 | Triose phosphate isomerase-R | GCACAATTGGCTGCATTTA |
| 13 | SORBIDRAFT_03g034200-F | GTATTATGGGTCCCAACTATGT |
| 14 | SORBIDRAFT_03g034200-R | CTCAACAGACTCTTGTTTCTTTC |
| 15 | NPRD-F | ACTCAGAGGCAATCTTATTTCT |
| 16 | NPRD-R | ATCGGGATTGAACTTCCTTAAT |
| 17 | Ubiquitin-F | CTCGCCGACTACAACATCC |
| 18 | Ubiquitin-R | AGGGCATCACAATCTTCACA |

**Supplemental Table S2** Reproducibility of 2-D gels

| **Sample** | **Average no of spots^a^** | **High quality spots^b^** | **Reproducibility (%)** |
| --- | --- | --- | --- |
| Control | 434 | 416 | 94.3 |
| Day 1  Day 3  Day 7 | 470  489  433 | 444  456  397 | 94  93  92.5 |

a = Average number of spots present in three replicate gels of each time point.

b = Spots having quality score more than 30 assigned by PDQuest (Ver.7.2.0).
